# Combined Effects of the Mobile Health (mHealth) Psychoeducation and Benson Relaxation Technique in Reducing the Caregiving Burden of Cancer Patients in Bangladesh: A Protocol for a Randomized Controlled Trial

**DOI:** 10.7759/cureus.55520

**Published:** 2024-03-04

**Authors:** Md Marufur Roshid, Md Moshiur Rahman, Md Nazmul Alam, Bilkis Banu, Kaniz Fateema Eity, Rafiur Rahman Shahin, Syeda Sabrina Easmin Shaba, Md Jiaur Rahman, Mohammad Habibur Rahman Sarker, Hitoshi Okamura

**Affiliations:** 1 Department of Psychosocial Rehabilitation, Graduate School of Biomedical and Health Sciences, Hiroshima University, Hiroshima, JPN; 2 Department of Health Sciences, Graduate School of Biomedical and Health Sciences, Hiroshima University, Hiroshima, JPN; 3 Department of Oncology, Khwaja Yunus Ali Medical College and Hospital, Sirajganj, BGD; 4 Department of Public Health, Northern University, Dhaka, BGD; 5 Department of Public Health and Health Policy, Graduate School of Biomedical and Health Sciences, Hiroshima University, Hiroshima, JPN; 6 Department of Nutrition and Clinical Service, International Centre for Diarrhoeal Disease Research (ICDDR), Dhaka, BGD

**Keywords:** bangladesh, cancer, female caregivers, informal caregivers, benson relaxation technique, psychoeducation, mhealth, caregiver burden

## Abstract

Background: Chronic and noncommunicable diseases, including cancer, are a significant global public health concern. Family members or friends who serve as caregivers significantly contribute to supporting cancer patients without formal medical training. In most cases in Bangladesh, women perform caregiving activities with household responsibilities and lack adequate support from the family and healthcare systems; consequently, they face a significant burden as caregivers. This study aims to assess the effectiveness of combined mobile health (mHealth) psychoeducation and the Benson relaxation technique (BRT) on the caregiving burden among female informal caregivers of cancer patients in Bangladesh.

Methods: We shall conduct a prospective, open-label, two-arm (1:1), randomized controlled trial in a hospital, focusing on the burden of informal female caregivers of cancer patients in Bangladesh. The combined intervention will be delivered to the intervention group through mHealth starting April 2024 and will span six months. Participants' data will be collected through face-to-face interviews using the Zarit Burden Interview (ZBI), the Hospital Anxiety Depression Scale, and the World Health Organization Quality of Life Bangla Short Instrument. Outcomes will be assessed at the baseline, midline, and endline. We shall employ descriptive statistics such as frequencies, percentages, means, and standard deviations. The t-test or Mann-Whitney U test will be used to compare continuous variables. Additionally, a two-way repeated-measures analysis of variance will be employed to evaluate the outcomes.

Results: Participant enrollment began in January 2024, and recruitment is ongoing. The results of this study will be disseminated through publications and conferences. No external professional writers were involved in writing this manuscript.

Conclusion: This study addresses the gap in the assessment of combined interventions for caregiver burden in Bangladesh. These outcomes may provide valuable insights into caregivers' well-being, caregiving responsibilities, and the potential for integrated interventions to reduce the burden, especially among women. If effective, we recommend the national integration of psychoeducation and BRT using mHealth.

## Introduction

Chronic and noncommunicable diseases represent major challenges of the 21st century, which ultimately result in severe consequences and societal issues for patients and communities worldwide [1]. Cancer, which claims the lives of more than 9.6 million people annually, 70% of whom are from lower- and middle-income countries, is a significant public health concern worldwide [2]. Cancer significantly affects patients, their families, and caregivers. In most cases, family members or close friends play a crucial role as informal caregivers in supporting and assisting cancer patients; these caregivers usually have no knowledgeable medical training [3]. The role of these caregivers is crucial throughout the disease trajectory of patients with cancer, that is, from diagnosis to the final stages of the illness. In the context of cancer, caregivers commonly face significant challenges related to patient treatment, symptom management, and the fulfillment of daily care needs. Consequently, it usually increases the levels of caregiving burden, anxiety, and depression and reduces the quality of life (QOL) that caregivers encounter throughout their caregiving journey [4,5].

The prevalence of caregiving burden among caregivers of cancer patients is significant and well-established. A study in India found that 92.5% of caregivers experienced mild-to-severe levels of burden [6]. Furthermore, a systematic review of various studies published on cancer nursing indicated that between 37% and 100% of family caregivers reported experiencing a certain level of burden [7]. Caregiving burden is negatively correlated with depression and anxiety [8]. Another systematic review highlighted that the prevalence of depression and anxiety among cancer caregivers was 42% and 47%, respectively [9]. The same study indicated that nearly half of the caregivers experienced severe levels of depression (46%) and anxiety (53%). Additionally, caregiving demands can result in physical, emotional, and social limitations, resulting in reduced overall QOL [10]. A previous study reported that the caregiving burden negatively impacts the QOL of caregivers of cancer patients [11].

Over the past few years, there has been a growing interest in utilizing digital health technologies, particularly mobile health (mHealth). As defined by the World Health Organization (WHO), "mHealth is a term used for medical and public health practices supported by mobile devices such as mobile phones, patient monitoring devices, personal digital assistants (PDAs), and other wireless devices" [12]. mHealth involves various communication channels such as short messaging services (SMS), phone calls, online platforms, social networks, and emails to provide interventions. mHealth psychoeducational interventions can equip caregivers with accessible information, self-care tools, and resources specific to their caregiving journey, which can improve their knowledge, skills, and self-efficacy, and ultimately enhance their capacity to manage caregiving responsibilities and their well-being [13,14]. One study reported the effectiveness of mHealth psychological interventions as a viable treatment approach for caregivers who experience high levels of stress or burden [15]. Relaxation techniques are nonpharmacological methods used to relieve mental health problems among patients and their caregivers. Among the various relaxation techniques available, the Benson relaxation technique (BRT) is considered one of the most effective, low-cost, and straightforward methods of intervention [16]. These techniques include mindfulness exercises that specifically treat a variety of mental and emotional symptoms such as burden, anxiety, pain, depression, mood swings, and self-esteem. Related studies have demonstrated that BRT is more effective in reducing caregiving burden, anxiety, and depression in various populations, including among caregivers [17,18].

In Bangladesh, there are 1.3-1.5 million cancer patients, among 0.2 million newly diagnosed patients every year [19]. Most female caregivers play a vital role in personally supporting patients. As a cultural expectation, women are generally expected to perform caregiving activities while maintaining household responsibilities and familial commitments, which places an additional burden on them. Females in Bangladesh experience the greatest disadvantages and vulnerabilities for numerous reasons. Research shows that compared with their male counterparts, female caregivers experience higher levels of burden and emotional distress, which significantly affects their QOL [10,11]. However, the unique cultural context, gender inequalities, and social norms in Bangladesh may cause additional difficulties for female caregivers. Therefore, it is essential to create effective interventions and support systems to alleviate their burden and improve their mental health and overall QOL. To the best of our knowledge, although there is growing evidence supporting the effectiveness of BRT and mHealth psychoeducational interventions separately, there is a significant knowledge gap, and their combined impact on informal female caregivers of cancer patients remains relatively unexplored. This research protocol following the PICOT (Population, Intervention, Comparison, Outcome, Time) framework outlines a randomized controlled trial aimed at evaluating the effectiveness of integrating an mHealth psychoeducational intervention and BRT in reducing the negative effects of the burden of caring for cancer patients in Bangladesh. This structured framework addresses specific research questions as follows: In female informal caregivers of cancer patients in rural Bangladesh (population), the combined mHealth psychoeducational intervention and Benson relaxation technique (intervention), compared with standard care (comparison), result in a reduction in caregiver burden, anxiety, and depression and an improvement in QOL (outcome) over a six-month period (time).

The results of this study suggest that the integration of mHealth psychoeducation and BRT is effective in reducing caregiver burden, increasing psychological well-being, and improving overall QOL.

## Materials and methods

Design and study site

**Figure 1:**
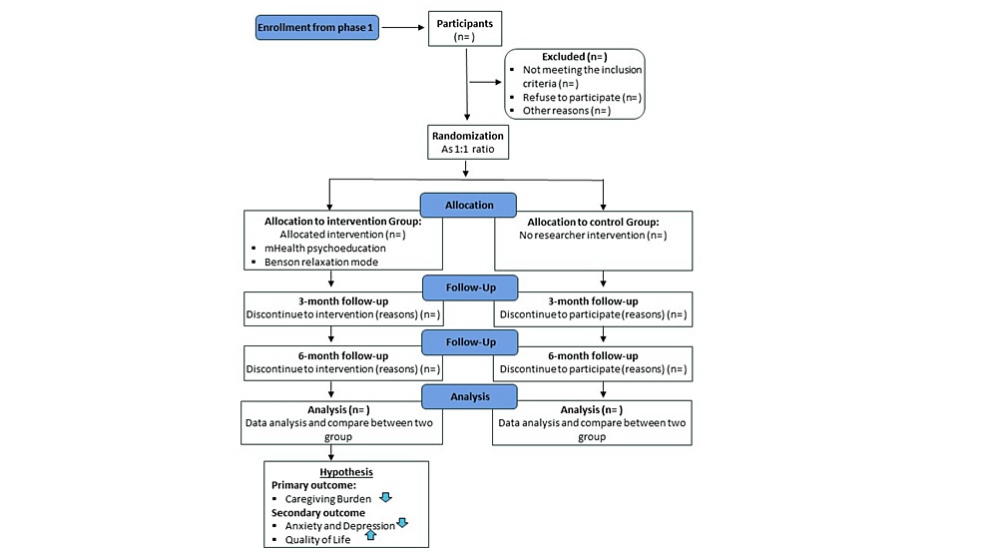
Flowchart of the study

This study is a hospital-based, single-centered, prospective, open-label, two-arm (1:1), randomized controlled trial involving the burden experienced by the caregivers of cancer patients and will be conducted in the rural areas of Bangladesh. This research is structured following the Consolidated Standards of Reporting Trials (CONSORT) and adheres to the SPIRIT (Standard Protocol Items: Recommendations for Interventional Trials) guidelines [20,21]. The intervention is scheduled to begin in April 2024, with the entire study spanning six months. Figure 1 shows a flowchart of the study. This study will be conducted at the Khwaja Yunus Ali Medical College and Hospital, located in Enayetpur, Sirajganj, Bangladesh. This hospital is recognized as a specialized cancer center in the northwestern part of the country. The study site has been selected purposefully because of the convenience of the research objectives.

Study participants

Eligibility Criteria

Initially, a baseline study was conducted to assess the caregiving burden among female informal caregivers of cancer patients. Subsequently, in this study, individual caregivers are enrolled based on their experiences with mild-to-moderate, moderate-to-severe, and severe burdens, as determined using the Bangla version of the Zarit Burden Interview (ZBI)-22 [22]. The inclusion criteria were as follows: females aged 18 years or older and those who have been providing care to a cancer-diagnosed patient for at least six months. The exclusion criteria included those who refused to collaborate with the ongoing study, caregivers receiving financial compensation or employed as formal caregivers, those experiencing extremely traumatic events that occurred during the study (such as the deaths of loved ones or patients, separations, etc.), caregivers undergoing psychotropic drug treatment, and those without smart mobile devices at home.

Sample Size

We used the G*Power software (Version 3.1.9.7) to calculate the sample size. An effect size of 0.72 was estimated based on a similar previous study that examined the caregiving burden among family caregivers [23]. The sample size required for the study was 84 caregivers, based on a power analysis of the difference between two independent means (two groups), a confidence level of 0.95, and a statistical power of 0.90. Eighteen additional caregivers will be recruited to compensate for potential dropouts. Following a 20% dropout rate, the total number of participants was 102, 51 in the intervention group (IG) and 51 in the control group (CG).

Data Collection and Follow-Up

Data will be collected at baseline (before the intervention), midline (after three months of intervention), and endline (after six months of intervention), through face-to-face interviews with participants. Sociodemographic data will be collected from two categories: caregivers and patients. Patients' basic characteristics such as sex, age, education, and marital status will be obtained directly from the patients. Additionally, relevant cancer-related information including cancer stage, type of cancer, duration of cancer, medical or other types of insurance coverage, and treatment type will be collected from hospital records. Data on caregivers' sociodemographic characteristics includes age, marital status, education level, employment status, monthly family income, presence of chronic diseases, relationship with the patient, duration of caregiving (months), and daily caregiving time (hours). Moreover, the ZBI-22 [22], Hospital Anxiety Depression Scale (HADS) [24], and World Health Organization Quality of Life Instrument (WHOQOL-BREF) [25] will be used to assess the level of burden, anxiety, depression, and QOL, respectively, all of which were validated and adopted in the local language, Bengali.

Randomization

After computer-generated stratification, participants will be randomly assigned according to their degree of burden, anxiety, and depression. In this study, there will be a 1:1 open-label randomization for either the IG or the CG. Randomization will be performed by a trained research assistant not involved in the intervention. The researchers will start organizing the study as soon as the research assistant informs them which participants are assigned to the IG or CG.

Intervention

In the IG, we designed a combined intervention using self-determination theory (SDT) and the BRT, which were used separately in previous studies, and successfully reduced the severity of the caregiving burden among caregivers [17,26]. There will be six sessions over six months, where each session will be conducted at the beginning of each month. During each session, videos recorded on the topic will be provided to the IG face-to-face, using smartphones, tablets, or handheld computers. Phone calls and SMS reminders will continue every week for follow-up and question-and-answer sessions. SDT will be employed for symptom management, maintaining and enhancing relationships, problem-solving, stress and coping, self-care, and effective communication. According to SDT principles, the symptom management component will provide education and resources to caregivers about symptom identification, monitoring, and management strategies appropriate for the specific needs of cancer patients, promoting the importance of social connections and supportive relationships to maintain and improve the care recipient and caregiver relationship. Caregivers will receive problem-solving skills training to identify and evaluate potential solutions, implement effective problem-solving strategies, and adjust their approach based on feedback and results. The intervention will provide educational materials and stress reduction techniques to help caregivers manage stress and improve their well-being. It means caregivers learn about how stress affects their bodies and minds and then practice various coping strategies to deal with the challenges of their caregiving role, promoting self-care practices to prioritize their own physical, emotional, and spiritual needs through activities such as exercising, relaxing, recreating, and self-reflection. The intervention included skills training to improve caregiver-patient communication and facilitate shared decision-making as part of effective communication based on self-determination theory. Definitions, benefits, and instructions regarding the BRT, such as diaphragmatic breathing, progressive muscle relaxation, and visualization exercises, will be provided through recorded videos. A nurse will guide the individuals face-to-face, after receiving training from a physiotherapist. Table 1 shows the intervention activities.

**Table 1:** Study activities BRT: Benson relaxation technique; SMS: short messaging services

Schedule	Intervention group	Control group
First month	First week: We will provide videos on information on cancer, symptom management, and the BRT with individual face-to-face training on the BRT by a trained nurse who will receive training from a physiotherapist. Second, third, and fourth weeks: Phone call and SMS reminder (once a week) for follow-up and a question-and-answer session.	Usual care
Second month	First week: We will provide a video on maintaining and enhancing relationships. Second, third, and fourth weeks: Phone call and SMS reminder (once a week) for follow-up and a question-and-answer session.	Usual care
Third month	First week: We will provide a video on problem-solving. Second, third, and fourth weeks: Phone call and SMS reminder (once a week) for follow-up and a question-and-answer session.	Usual care
Fourth month	First week: We will provide videos on stress and coping. Second, third, and fourth weeks: Phone call and SMS reminder (once a week) for follow-up and a question-and-answer session.	Usual care
Fifth month	First week: We will provide a video on self-care. Second, third, and fourth weeks: Phone call and SMS reminder (once a week) for follow-up and a question-and-answer session.	Usual care
Sixth month	First week: We will provide a video on effective communication. Second, third, and fourth weeks: Phone call and SMS reminder (once a week) for follow-up and a question-and-answer session.	Usual care

Endpoints

The primary outcome involves assessing the change scores obtained from the ZBI. Meanwhile, the secondary outcomes are as follows: (1) assessment of changes in anxiety and depression levels by the HADS and (2) changes in QOL, as measured by the WHOQOL questionnaire. Both primary and secondary outcomes will be assessed at baseline, midline, and endline in both the IG and CG.

Measurements

ZBI

As a measure of caregiver burden, we will use the ZBI, which consists of 22 items [22]. The frequency of experiencing certain caregiver-related challenges is expressed on a Likert scale ranging from 0 to 4 as follows: 0=never, 1=rarely, 2=sometimes, 3=quite frequently, and 4=nearly always. Where higher scores represent a higher burden, the scores are the following: 0-21=little or no burden, 21-40=mild-to-moderate burden, 41-60=moderate-to-severe burden, and 61-88=severe burden.

HADS

The HADS will be used to evaluate the anxiety and depression levels [24]. Among the 14 items on a scale from 0 to 3, higher scores indicate anxiety or depression and the severity of their experience. Anxiety and depressive symptoms will be categorized according to predetermined criteria as follows: 0-7=normal levels, 8-10=borderline abnormal levels, and 11-21=abnormal levels.

WHOQOL-BREF

The WHOQOL-BREF will be used to assess the caregiver's QOL [25]. This instrument is particularly designed to evaluate multiple QOL components, including social interactions, environmental variables, psychological well-being, and physical health. A total of 26 items will rate their experiences on a Likert scale ranging from 1 to 5.

Data safety monitoring plan

Participants in this study have no minimal risk, and the researcher will treat all their personal information, including names, phone numbers, and addresses, as confidential and keep them secure. Confidentiality procedures will be implemented to ensure that participants' information is kept secure. Only members of our survey research team will have access to the data obtained from the questionnaires.

Statistical analysis

The IBM SPSS Statistics for Windows, Version 25.0 (Released 2017; IBM Corp., Armonk, New York, United States) will be used to evaluate the data. The results of the IG and CG will be compared using an intention-to-treat analysis. Continuous data will be presented as mean and standard deviation (SD), while categorical data will be represented as frequencies and percentages. Baseline data will be analyzed using various statistical tests, such as the chi-squared test, t-test, Mann-Whitney U test, Pearson's X2 test, or Fisher's exact test, to compare the two groups (IG and CG). A specific test will be employed depending on the variable type and distribution of the data. The mean values of the ZBI, HADS, and WHOQOL-BREF will be compared between the two groups at baseline, midline, and endline to assess the efficacy of the intervention. Comparisons will be performed using independent t-tests. Additionally, two-way repeated-measures analysis of variance (ANOVA) will be employed to evaluate changes in outcomes over time within each group (from baseline to endline). This analysis considers the IG, time, and their interactions. Statistical significance will be set at p<0.05. The latest observation carried forward approach will be applied to data imputation to resolve missing data.

Ethical considerations

The principles of the Declaration of Helsinki will be followed while conducting the study [27]. We obtained ethical approval from the Ethical Review Committee (ERC) of the Department of Public Health, Northern University Bangladesh (NUB) (approval number: NUB/DPH/EC/2023/26), and the clinical trial has been registered on ClinicalTrials.gov under the clinical trial registration number NCT06204328.

## Results

The enrollment of study participants began in January 2024 and is ongoing. The principal investigators will meet near the end of the trial to complete and execute the research dissemination plan and authorship details. The findings will be shared with the research community, policymakers, and health planners through peer-reviewed publications and conference presentations. We do not intend to include paid professional writers outside the study team. Participants will be informed of the results individually at the end of the trial, and interventional materials will be provided to the control group. This intervention is expected to begin in April 2024.

## Discussion

Zarit et al. define caregiver burden as the range of physical, psychological, social, and financial reactions that can occur while giving care [28]. This study employs a psychoeducational intervention as well as the BRT, focusing on promoting self-determination. The aim is to reduce the range of physical, psychological, and social reactions among caregivers, which is expected to be achieved by increasing caregivers' knowledge of symptom management, maintaining and enhancing relationships, problem-solving, stress and coping, self-care, and effective communication for cancer patients.

Furthermore, several studies have demonstrated that the utilization of mHealth increases knowledge, skills, and self-efficacy regarding caregiving responsibilities and the ability to manage one's well-being [13,14]. These platforms offer a promising solution for delivering psychoeducational content support to caregivers in low-resource settings like Bangladesh, where traditional healthcare access is limited. mHealth technology can empower caregivers with convenient access to educational resources and relaxation techniques, which will be an additional advantage in increasing their knowledge, efficacy, and capability to manage the demands of caregiving.

Individually, the effectiveness of mHealth psychoeducation and BRT on caregiver burden has been shown to be positive in several studies, whereas some have demonstrated no significant impact [29,30]. After recognizing the importance of sociocultural context, this study prioritizes the need for effective interventions for caregivers in Bangladesh. We expect that the combined intervention will enhance caregivers' knowledge and self-confidence, resulting in a reduction in burden and an improvement in mental health and overall QOL. These positive outcomes will contribute significantly to overall care, potentially leading to increased life expectancy among cancer patients.

To the best of our knowledge, this study represents the first research endeavor in Bangladesh to evaluate the outcome of combined interventions of psychoeducation and the BRT through mHealth, to reduce the caregiving burden for informal Bangladeshi female caregivers of cancer patients. Additionally, our study will provide information on the levels of anxiety and depression as well as the contributing factors among female informal caregivers of cancer patients in Bangladesh. Moreover, this study will estimate the position of QOL and its associated factors among female caregivers. Our study has several limitations. We expect that 102 caregivers will be recruited for the interventions. This depends on the circumstances at the study site. The patients of recruited caregivers will have different types of cancer, including those at different stages, socioeconomic statuses, and education levels, which may affect our results.

## Conclusions

Our study provides potential insights into general well-being, caregiving responsibilities, and the ability to manage one's well-being through combined interventions, to reduce caregiver burden, especially among women. Furthermore, this strategy may emphasize the improvement of mental health and QOL. If our findings indicate positive outcomes among caregivers experiencing burden, we recommend the integration of psychoeducation and the BRT through mHealth as an effective strategy at the national level.
